# A Damaged Oxidative Phosphorylation Mechanism Is Involved in the Antifungal Activity of Citral against *Penicillium digitatum*

**DOI:** 10.3389/fmicb.2018.00239

**Published:** 2018-02-16

**Authors:** Qiuli OuYang, Nengguo Tao, Miaoling Zhang

**Affiliations:** School of Chemical Engineering, Xiangtan University, Xiangtan, China

**Keywords:** *Penicillium digitatum*, citral, iTRAQ, oxidative phosphorylation, reactive oxygen species

## Abstract

Citral exhibits strong antifungal activity against *Penicillium digitatum*. In this study, 41 over-expressed and 84 repressed proteins in *P. digitatum* after 1.0 μL/mL of citral exposure for 30 min were identified by the iTRAQ technique. The proteins were closely related with oxidative phosphorylation, the TCA cycle and RNA transport. The mitochondrial complex I, complex II, complex III, complex IV and complex V, which are involved in oxidative phosphorylation were drastically affected. Among of them, the activities of mitochondrial complex I and complex IV were apparently suppressed, whereas those of mitochondrial complex II, complex III and complex V were significantly induced. Meanwhile, citral apparently triggered a reduction in the intracellular ATP, the mitochondrial membrane potential (MMP) and glutathione content, in contrast to an increase in the glutathione S-transferase activity and the accumulation of reactive oxygen species (ROS). Addition of exogenous cysteine decreased the antifungal activity. In addition, cysteine maintained the basal ROS level, deferred the decrease of MMP and the membrane damage. These results indicate that citral inhibited the growth of *P. digitatum* by damaging oxidative phosphorylation and cell membranes through the massive accumulation of ROS.

## Introduction

The green mold caused by *Penicillium digitatum* is a damaging disease in citrus fruits (Jing et al., 2014). Currently, this disease is mainly controlled by the intensive use of synthetic fungicides, but the application of chemical fungicides usually leads to the appearance of resistant strains and brings concerns about food and environmental safety. Plant essential oils and their volatile components are attracting considerable research efforts because of their potential use as food preservatives and additives in controlling postharvest diseases in fruits (Pérez-Alfonso et al., 2012; Shao et al., 2015; Tian et al., 2015; Boubaker et al., 2016; Li Y. H. et al., 2016).

Citral (3,7-dimethyl-2,6-octadienal) is mixture of two isomers, namely, geranial and neral, and is extracted from several lemon-scented herbal plants, most notably lemons, verbena, and lemongrass. Because of its particular aroma, substantial antibacterial, antifungal and insecticidal effects, as well as its low toxicity and low carcinogenicity, citral is classified as a substance that is “Generally Recognized as Safe” (GRAS) and has been widely used as a food additive and fragrance material in cosmetics. In recent years, citral has been illustrated to exhibit strong antifungal activities against *P. digitatum, P. italicum*, and *Geotrichum citri-aurantii* (Wuryatmo et al., 2003, 2014; Tao et al., 2014a; Zhou et al., 2014). In our previous studies, citral was found to inhibit the mycelial growth of *P. digitatum* in a dose dependent manner with a minimum inhibitory concentration (MIC) of 2.0 μL/mL and a minimum antifungal concentration (MFC) of 4.0 μL/mL, and the wax + citral (10 × MFC) treatment significantly decreased the incidence of green mold in Ponkan mandarin fruit after 6 days of storage at 25 ± 2°C, but did not influence the external and internal fruit qualities of citrus fruit (Fan et al., 2014). Therefore, citral is a promising substance that can be used in biological control of postharvest diseases in citrus fruit.

The potential mechanisms underlying the antifungal activity of citral are not fully understood, but several possible mechanisms have been proposed. The lipophilic nature of citral enables it to have the capacity to permeabilize the cell membrane, disrupt cell integrity, cause the leakage of cellular components, and finally lead to the cell death (Harris, 2002). Park et al. (2009) demonstrated that the cell membrane and organelles of *Trichophyton mentagrophytes* were irreversibly damaged by 0.2 mg/ml citral. Rajput and Karuppayil (2013) found that citral could exert their antifungal effect through inhibition of ergosterol biosynthesis. In a recent study, citral was able to alter the morphology of *Candida albicans*, but did not influence the cell wall or ergosterol (Leite et al., 2014). We previously reported that citral could inhibit the mycelial growth of *P. digitatum, P. italicum*, and *G. citri-aurantii* by a membrane damage mechanism (Tao et al., 2014b; Zhou et al., 2014; OuYang et al., 2016a). Meanwhile, citral evidently altered the mitochondrial morphology and repressed the citrate cycle (TCA cycle), respiratory metabolism and glycolysis of *P. digitatum* (Tao et al., 2015; Zheng et al., 2015). RNA-Seq data further showed that citral treatment greatly affected the expression levels of genes participating in ABC transport, steroid biosynthesis, the TCA cycle, oxidative phosphorylation, RNA degradation and ribosome biosynthesis (OuYang et al., 2016b).

It is well known that proteins serve an indispensable role in mediating the adaptability of pathogens to different stresses (Lackner et al., 2012), and proteins involved in the interaction of the pathogen with fungicides are crucial to understand the inhibition mechanism of fungicides on pathogens. In recent years, several techniques were developed aiming at understanding the complex biological systems and determining the relationships between proteins, their functions, and protein–protein interactions, such as isobaric tags for relative and absolute quantitation technique (iTRAQ), two-dimensional polyacrylamide gel electrophoresis and two-dimensional difference gel electrophoresis (Zieske, 2006). Among of them, iTRAQ is becoming one of the most powerful tools to compare the protein expression patterns in microorganisms under different condition (Redding et al., 2006; Taylor et al., 2008; Yang et al., 2015; Zhang et al., 2015; Liu et al., 2017). To the best of our knowledge, however, the research considering the *P. digitatum* proteome in response to citral has not been conducted until now. Therefore, this research aims to identify differentially expressed proteins (DEPs) in *P. digitatum* upon exposure to citral by iTRAQ, in an effort to elucidate the antifungal mechanism of citral on *P. digitatum*.

## Materials and methods

### Fungal strains

*P. digitatum* was isolated from infected citrus fruit and preserved on potato dextrose agar at 25 ± 2°C. Two hundred micro liter fungal suspensions (5 × 10^5^ cfu/mL) were added to the 40-mL potato dextrose broth (PDB) and incubated in a moist chamber at 25 ± 2°C. The mycelia of *P. digitatum* grown for 48 h were collected and re-suspended in phosphate buffered saline (pH 7.0). The suspensions were then treated with 1/2MIC (1.0 μL/mL) of citral and incubated at 25 ± 2°C under agitation in an environmental incubator shaker for 30 min (OuYang et al., 2016b). The resulting samples were selected and named T30. The mycelia treated with phosphate buffered saline (pH 7.0) for 30 min were used as a negative control (CK30). All *P. digitatum* mycelia were immediately frozen in liquid nitrogen and stored at −80°C until further analysis.

### Quantitative proteomics by iTRAQ and LC-ESI-MS/MS

Total proteins were extracted from the mycelia as described by Zhang et al. (2015) The samples were solubilized with 500 mM triethylammonium bicarbonate (TEAB) and quantified by the Bradford assay (Bradford, 1976). One hundred micro grams of proteins were taken out of each sample solution and digested with Trypsin Gold (Promega, Madison, WI, USA) with a protein:trypsin ratio of 30:1 at 37°C for 16 h. After trypsin digestion, peptides were dried by vacuum centrifugation. Peptides were reconstituted in 0.5 M TEAB and processed according to the manufacture's protocol for 8-plex iTRAQ reagent (Applied Biosystems, Milan, Italy). Briefly, one unit of iTRAQ reagent was thawed and reconstituted in 24 μL isopropanol. Samples were labeled with the iTRAQ tags 117, 119, 114, and 115. The peptides were labeled with the isobaric tags, incubated at room temperature for 2 h. The labeled peptide mixtures were then pooled and dried by vacuum centrifugation. The labeled peptides were separated by SCX chromatography with a LC-20AB HPLC Pump system (Shimadzu, Kyoto, Japan) as described previously (Zhang et al., 2015).

After reconstituting dried fractions with solvent A (5% ACN and 0.1% FA) to a concentration of 0.5 μg/μL, 5 μL samples were analyzed on an LC-20AD nano-LC-ESI-MS/MS system (Shimadzu, Kyoto, Japan), and data acquisition was performed with a TripleTOF 5600 System (AB SCIEX, Concord, ON) fitted with a Nanospray III source (AB SCIEX, Concord, ON) and a pulled quartz tip as the emitter (New Objectives, Woburn, MA) (Zhang et al., 2015).

### Data analysis and bioinformatics analysis

For protein identification, a mass tolerance of 0.05 Da (ppm) was permitted for intact peptide masses and 0.1 Da for fragmented ions, allowing for one missed cleavage in the trypsin digests. Gln- > pyro-Glu (N-term Q), oxidation (M), deamidated (NQ) were treated as potential variable modifications, and carbamidomethyl (C), iTRAQ8plex (N-term), iTRAQ8plex (K) as fixed modifications. The charge states of the peptides were set to +2 and +3. Specifically, an automatic decoy database search was performed in Mascot by choosing the decoy checkbox in which a random sequence database is generated and tested for raw spectra as well as the real database. To reduce the probability of false peptide identification, only peptides with significance scores (≥20) at the 99% confidence interval by a Mascot probability analysis greater than the “identity” were counted as identified. Each confident protein identification involves at least one unique peptide.

For protein quantization, it was required that a protein contains at least two unique peptides. The quantitative protein ratios were weighted and normalized by the median ratio in Mascot. We only used ratios with *p* < 0.05, and only fold changes of > 1.5 were considered significant.

Functional annotations of the proteins was conducted using the Blast2GO program against the non-redundant protein database (NR; NCBI). The KEGG database (http://www.genome.jp/kegg/) and the COG database (http://www.ncbi.nlm.nih.gov/COG/) were used to classify and group these identified proteins, and then, the proteins were assigned to 108 known biological pathways in the Kyoto Encyclopedia of Genes and Genomes (KEGG) database (www.genome.jp/kegg/).

### Enzymatic activities of mitochondrial respiratory complexes

The enzymatic activities of the mitochondrial complex I, complex II, complex III, complex IV, and complex V of the *P. digitatum* cells with citral at 0 and 1/2MIC for 0, 30, 60 and 120 min were determined by a UV2450 UV/Vis spectrophotometer [Shimadzu (Shanghai), Shanghai, China] using the commercially available kits (Solarbio Beijing, Beijing, China) following the manufacturer's instructions. Three independent replicates were performed for each treatment.

### Glutathione S-Transferase (GST) activities and glutathione (GSH) contents

The GST activities and GSH contents of *P. digitatum* cells with or without 1/2MIC of citral for 0, 30, 60, and 120 min were determined by a UV2450 UV/Vis spectrophotometer using a commercially available kit (Solarbio Beijing, Beijing, China) following the manufacturer's instructions. Three independent replicates were performed for each treatment.

### ATP contents

The intracellular and extracellular ATP contents of *P. digitatum* cells treated with 1/2MIC of citral or not were determined according to our previous method (Zheng et al., 2015).

### Mitochondrial membrane potential (MMP)

*P. digitatum* hyphae incubated with 1/2MIC citral or without citral for 0, 30, 60, and 120 min were used to determine the MMP following the JC-10 Assay Kit (Solarbio, Shanghai, China). The treated cells were stained with JC-10 and analyzed with an ECLIPSE TS100 microscope (Nikon, Japan). The fluorescence values were measured by a F97 PRO fluorescence spectrophotometer (Lengguang Technology, Shanghai, China). Three replications for each treatment were performed. These experiments were also performed in samples supplied with antioxidant cysteine (Cys) at a concentration of 10 μM.

### Reactive oxygen species (ROS) levels

The ROS levels in *P. digitatum* cells treated with citral at 1/2MIC or not for 0, 30, 60, and 120 min were determined by a redox-sensitive fluorescent probe dichloro-dihydro-fluorescein diacetate (DCFH-DA), according to the ROS assay kit (Solarbio Beijing, Beijing, China) instructions. The fluorescence values were measured by a F97 PRO fluorescence spectrophotometer (Lengguang Technology, Shanghai, China). Three independent replicates were performed for each treatment. The mycelia were observed with an ECLIPSE TS100 microscope (Nikon, Japan). These experiments were also conducted in samples supplied with the antioxidant Cys at a concentration of 10 μM.

### Effect of exogenous cys on the antifungal activity of citral against *P. digitatum*

Cys at a final concentration of 10 μM was added to the *P. digitatum* cultures supplied citral at the concentrations 0.0 μL/mL, 1.0 μL/mL (1/2MIC), and 2.0 μL/mL (MIC). The antifungal activity was measured by the agar dilution method (Tao et al., 2014b). The cultures without Cys were used as the negative control.

### Plasma membrane integrity

Plasma membrane integrity of the *P. digitatum* cells with citral (0 or 1/2MIC) were analyzed by propidium iodide (PI) staining coupled with fluorescence microscopy (Liu et al., 2010) with minor modifications. The 2-day-old mycelia from 50 mL PDB were collected and centrifuged at 4,000 g for 10 min. The collected mycelia were stained with 10 μg/mL of PI for 15 min at 30°C. Residual dyes were removed by washing twice with PBS (pH 7.0). Samples were observed with an ECLIPSE TS100 microscope (Nikon, Japan), and the fluorescence value was determined by a F97 PRO fluorescence spectrophotometer (Lengguang Technology, Shanghai, China). These experiments were also performed with antioxidant Cys at a concentration of 10 μM.

### Statistical analysis

All data were expressed as the mean ± SD (standard deviation) by measuring three independent replicates and analyzed by one-way analysis of variance (ANOVA) followed by Duncan's multiple range test. A value of *P* < 0.05 was considered statistically significant, and data were analyzed using the SPSS statistical software package release 16.0 (SPSS Inc., Chicago, IL, USA).

## Results

### Proteins in *P. digitatum* cells

Based on the iTRAQ-labeled peptides, a total of 3,251 proteins were isolated or identified. These proteins were further categorized into three GO ontologies, including biological processes, cellular components, and molecular functions. The most favored biological process was the “metabolic process” (29.34%), mainly consisting of the “cellular process” (27.35%) and “single-organism process” (10.70%) subcategories. A greater number of proteins were assigned to the cellular component category. The largest subclasses of proteins within this group were “cell” (24.69%) and “cell part” (24.69%). Categorization of the identified proteins on the basis of molecular function indicated that the most abundant proteins belonged to “catalytic activity” (47.28%) and “binding” classes (41.09%) (Table S1).

These proteins were further classified into 24 COG functional subcategories (Table S2). Most of these proteins were involved in “general function prediction only” (515 proteins), “translation, ribosomal structure and biogenesis” (298 proteins), and “posttranslational modification, protein turnover, chaperones” (246 proteins). Only a few proteins were associated with “defense mechanisms” (17 proteins), “cell motility” (5 proteins), and “nuclear structure” (1 proteins). A total of 2,443 proteins were assigned to 107 KEGG pathways (Table S3). Among them, 708, 285, and 92 proteins were distributed to the metabolic pathway, biosynthesis of secondary metabolites, and purine metabolism, respectively. One protein that correlated with glycosaminoglycan degradation or caffeine metabolism was also found.

### Differentially expressed proteins induced by citral

The difference in the protein expressions between CK30 and T30 was further analyzed. As a result, 125 proteins, including 41 up-regulated and 84 down-regulated proteins, were identified as DEPs. Among them, 82 DEPs were mapped to the KEGG database and assigned to 42 specific pathways, such as oxidative phosphorylation, endocytosis, ribosome, spliceosome, RNA degradation, ribosome biogenesis in eukaryotes, peroxisome, MAPK signaling pathway, glutathione metabolism, and cell cycle (Table 1 and Table S4).

**Table 1:** Enrichment pathway analysis of DEPs in *P. digitatum*.

	**Pathway**	**Diff proteins with pathway annotation (82)**	**All proteins with pathway annotation (2,443)**	***P*-value**	**Pathway ID**
1	Oxidative phosphorylation	10 (12.2%)	86 (3.52%)	0.000461176	ko00190
2	Endocytosis	5 (6.1%)	47 (1.92%)	0.01912532	ko04144
3	Ribosome	7 (8.54%)	84 (3.44%)	0.02083807	ko03010
4	Caffeine metabolism	1 (1.22%)	1 (0.04%)	0.03356529	ko00232
5	SNARE interactions in vesicular transport	2 (2.44%)	13 (0.53%)	0.0683191	ko04130
6	Non-homologous end-joining	1 (1.22%)	4 (0.16%)	0.1277259	ko03450
7	Basal transcription factors	2 (2.44%)	22 (0.9%)	0.1671543	ko03022
8	Spliceosome	5 (6.1%)	90 (3.68%)	0.1821064	ko03040
9	Synthesis and degradation of ketone bodies	1 (1.22%)	6 (0.25%)	0.1854038	ko00072
10	Glyoxylate and dicarboxylate metabolism	2 (2.44%)	24 (0.98%)	0.1915471	ko00630
11	Nitrogen metabolism	2 (2.44%)	24 (0.98%)	0.1915471	ko00910
12	Pentose and glucuronate interconversions	2 (2.44%)	25 (1.02%)	0.2039195	ko00040
13	RNA degradation	3 (3.66%)	51 (2.09%)	0.243635	ko03018
14	Alanine, aspartate and glutamate metabolism	2 (2.44%)	31 (1.27%)	0.2793793	ko00250
15	Fatty acid metabolism	2 (2.44%)	31 (1.27%)	0.2793793	ko00071
16	Arginine and proline metabolism	2 (2.44%)	37 (1.51%)	0.3545094	ko00330
17	Ribosome biogenesis in eukaryotes	3 (3.66%)	64 (2.62%)	0.3644286	ko03008
18	Peroxisome	2 (2.44%)	38 (1.56%)	0.3668124	ko04146
19	Biosynthesis of unsaturated fatty acids	1 (1.22%)	14 (0.57%)	0.380771	ko01040
20	MAPK signaling pathway - yeast	2 (2.44%)	41 (1.68%)	0.4031635	ko04011

Nineteen proteins related with the ribosome, RNA degradation, ribosome biogenesis in eukaryotes, spliceosome, basal transcription factors, RNA transport and mRNA surveillance pathway were affected by citral (Table 2, Tables S5, S6). Interestingly, the 60S ribosomal protein L35, the 60S ribosomal protein L35Ae, and the ribosomal protein S24 involved in ribosome were up-regulated after citral treatment, while the ribosomal protein L44e, 40S ribosomal protein S26E, 60S ribosomal protein L28, and 60S ribosomal protein L38 were down-regulated.

**Table 2:** Translational-related DEPs and energy production and conversion-related DEPs in *P. digitatum*.

**Proteins**	**NCBInr accession**	**Change folds**
60S ribosomal protein L35	gi|425779472|gb|EKV17524.1|	1.562
60S ribosomal protein L35Ae	gi|425783374|gb|EKV21228.1|	1.426
Ribosomal protein L44e	gi|425766420|gb|EKV05032.1|	−0.707
40S ribosomal protein S24	gi|425773071|gb|EKV11444.1|	1.469
40S ribosomal protein S26E	gi|425769642|gb|EKV08131.1|	−0.727
60S ribosomal protein L28	gi|425782319|gb|EKV20238.1|	−0.694
60S ribosomal protein L38	gi|425782646|gb|EKV20545.1|	−0.801
Transcription initiation factor TFIID subunit TSM1/127kD	gi|425783990|gb|EKV21801.1|	−0.747
Hypothetical protein PDIP_22250 TAF9	gi|425781794|gb|EKV19739.1|	1.427
Small nuclear ribonucleoprotein SmG	gi|425783989|gb|EKV21800.1|	1.395
Hypothetical protein PDIP_32220 SF3b	gi|425779117|gb|EKV17206.1|	1.958
Small nuclear ribonucleoprotein SmG Lsm	gi|425783989|gb|EKV21800.1|	1.395
Splicing factor u2af large subunit	gi|425773483|gb|EKV11835.1|	−0.649
U2 auxiliary factor small subunit	gi|425770129|gb|EKV08603.1|	−0.818
MRNA decapping hydrolase DCPs	gi|425765840|gb|EKV04486.1|	1.448
Hypothetical protein PDIP_50840 PABP1	gi|425774597|gb|EKV12899.1|	−0.574
Small nuclear ribonucleoprotein (LSM7)	gi|425770497|gb|EKV08967.1|	1.412
Casein kinase II beta subunit CKB1	gi|425781873|gb|EKV19809.1|	−0.731
Hypothetical protein PDIP_22250 Fap7	gi|425781794|gb|EKV19739.1|	1.427
Acyl carrier protein	gi|425769175|gb|EKV07676.1|	−0.565
LYR family protein	gi|425784140|gb|EKV21934.1|	−0.596
NADH-ubiquinone oxidoreductase	gi|425775152|gb|EKV13435.1|	−0.692
Hypothetical protein PDIP_56530	gi|425773011|gb|EKV11388.1|	−0.785
Hypothetical protein PDIP_64010	gi|425771150|gb|EKV09603.1|	2.338
Cytochrome b-c1 complex subunit 6	gi|425778715|gb|EKV16822.1|	−0.607
Cytochrome c oxidase polypeptide vib	gi|425778405|gb|EKV16533.1|	−0.698
Cytochrome c oxidase copper chaperone Cox17	gi|425783196|gb|EKV21055.1|	−0.673
ATP synthase delta chain	gi|425766735|gb|EKV05334.1|	1.626
Glutamine synthetase	gi|425776031|gb|EKV14269.1|	−0.661
Electron transfer flavoprotein alpha subunit putative	gi|425773543|gb|EKV11891.1|	−0.691
Delta-1-pyrroline-5-carboxylate dehydrogenase PrnC	gi|425778146|gb|EKV16288.1|	−0.757
Succinate dehydrogenase cytochrome b560 subunit	gi|425773767|gb|EKV12100.1|	2.250
Glutathione S-transferase	gi|425768826|gb|EKV07338.1|	1.444

The expression pattern of the proteins related to energy and reactive oxygen species (ROS) were greatly influenced by the addition of citral (Table 2, Tables S5, S6). Approximately 12 mitochondrial proteins were found to be differentially expressed in response to 1/2MIC of citral. Among these DEPs, the succinate dehydrogenase (ubiquinone) flavoprotein subunit and GST, which were involved in the citrate cycle (TCA cycle) and glutathione metabolism, respectively, were both up-regulated. In addition, the rest of the enzymes that belong to oxidative phosphorylation that mainly participate in the electron transport chain and are located in the inner mitochondrial membrane were significantly affected by citral. For example, the following enzymes were down-regulated: acyl carreier protein (Ndufab1), LYR family protein (Ndufb9), NADH-ubiquinone oxidoreductase (Ndufs6) and hypothetical protein PDIP_56530 (Ndufb8), all of which constitute the mitochondrial complex I; cytochrome b-c1 complex subunit 6 (QCR6), which belongs to mitochondrial complex III; and cytochrome c oxidase polypeptide vib (COX6B) and cytochrome c oxidase copper chaperone Cox17 (COX17), which belong to mitochondrial complex IV. The following enzymes were up-regulated: succinate dehydrogenase cytochrome b560 subunit (SDHC), which belongs to mitochondrial complex II; hypothetical protein PDIP_64010 (QCR10), which belongs to mitochondrial complex III; and, ATP synthase delta chain, which belongs to mitochondrial complex V.

### Mitochondrial respiration complexes activities

**Figure 1:**
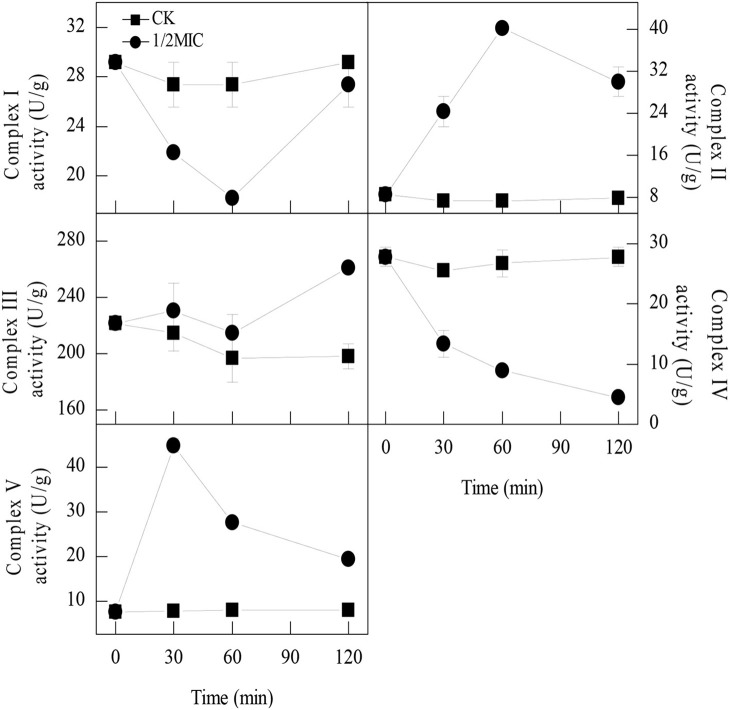
Activities of the five enzymes involved in oxidative phosphorylation of *P. digitatum* mycelia. Data presented are the means of the pooled data. Error bars indicate the SDs of the means (*n* = 3).

The enzymatic activities of the mitochondrial respiration complexes were found to be consistent with those of the iTRAQ analysis. The mitochondrial complex I activity in the control samples remained relatively stable during the entire period. In contrast, the mitochondrial complex I activity in the samples treated with 1/2MIC of citral was sharply decreased at 60 min of exposure, with the values reaching 18.3 ± 0.0 U/g, which was significantly lower than that of the control sample (27.4 ± 1.8 U/g, *P* < 0.05; Figure 1). After 120 min of exposure, the mitochondrial complex I activity in the *P. digitatum* cells treated with 1/2MIC of citral was notably increased to 27.4 ± 1.8 U/g and no obvious difference between the treated and untreated cells was observed. The mitochondrial complex II and complex V activities in the citral treated samples were quite different from those of mitochondrial complex I. The mitochondrial complex II and complex V activities increased to peak values (40.1 ± 0.8 U/g, 44.9 ± 1.5 U/g) at 60 and 30 min of exposure, respectively, which were significantly higher than the control (7.3 ± 0.6 U/g, 7.7 ± 0.7 U/g). At 120 min, mitochondrial complex II and complex V activities in 1/2MIC of citral treated groups dropped to 29.9 ± 2.8 U/g and 19.3 ± 0.4 U/g, respectively, which were still higher than the control sample (7.9 ± 0.0 U/g, 8.0 ± 1.1 U/g). In the case of the mitochondrial complex III, no difference in the activities between the 1/2MIC-treated samples and the control samples was observed before 60 min of exposure. After 120 min of exposure, the mitochondrial complex III activity in the control groups remained at a comparable level with the initial exposure, whereas its activity in 1/2MIC citral treated samples was induced and reached 261.0 ± 3.1 U/g. The mitochondrial complex IV activity was impaired by the addition of citral. Compared with the control samples, the mitochondrial complex IV activities in the 1/2MIC citral-treated samples decreased from 47.8 to 84.0% during citral exposure.

### GST activities and GSH contents

**Figure 2:**
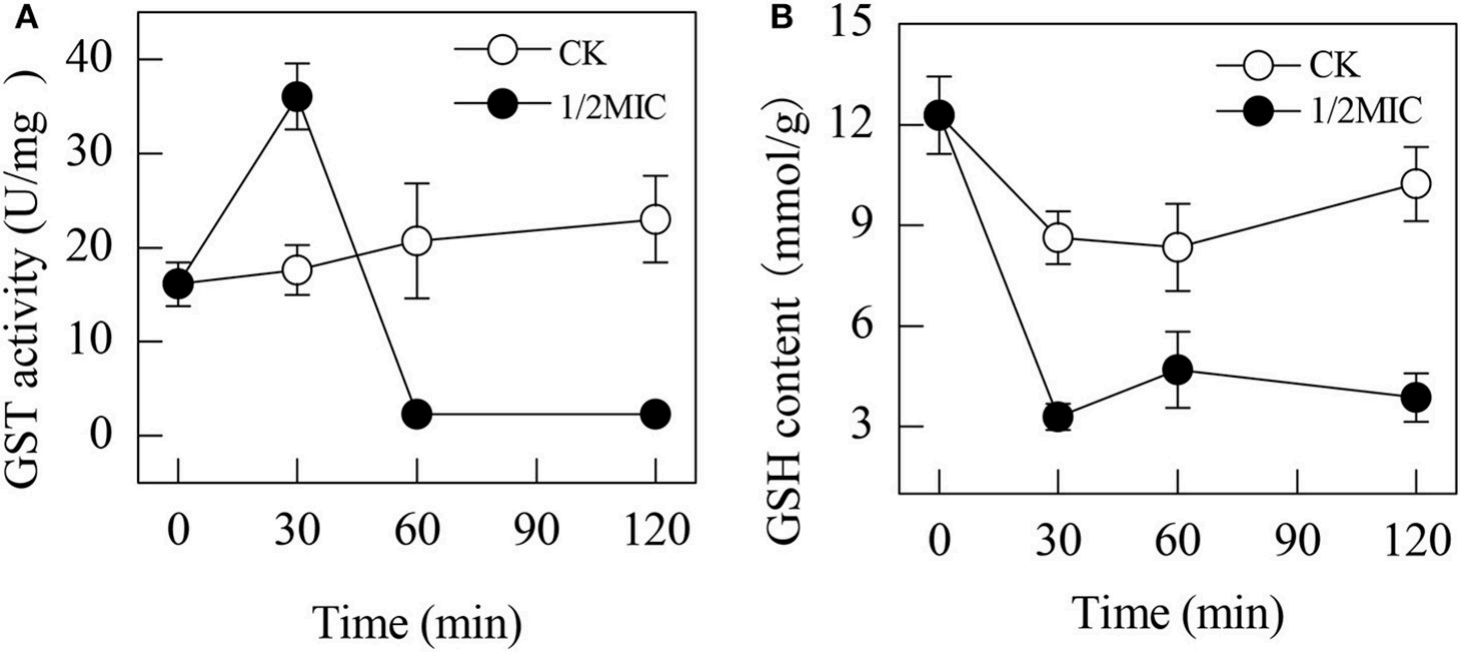
Effects of citral on the GST activities **(A)** and GSH contents **(B)** of *P. digitatum* mycelia. Data presented are the means of the pooled data. Error bars indicate the SDs of the means (*n* = 3).

The activity of GST was induced by the addition of citral during the initial 30 min (Figure 2A). At 30 min of exposure, the GST activity in the citral-treated sample was 36.03 ± 3.51 U/mg, which was higher than that of the control (17.63 ± 2.65 U/mg, *P* < 0.05). However, the activity of GST in *P. digitatum* cells with 1/2MIC of citral was suddenly decreased over the remaining period and remained at a lower level compared to that of the control samples (*P* < 0.05).

The GSH content in the control samples was significantly higher than those of the citral treated samples (Figure 2B). In contrast, the GSH content in the citral-treated *P. digitatum* was significantly decreased (*P* < 0.05) from 12.3 ± 1.2 μmol/g at the initial exposure to 3.2 ± 0.4 μmol/g at 30 min of exposure, which was significantly lower than that in the control samples. After 30 min of exposure, the GSH contents in the 1/2MIC-treated *P. digitatum* cells maintained a relatively low level throughout the whole period.

### ATP contents

**Figure 3:**
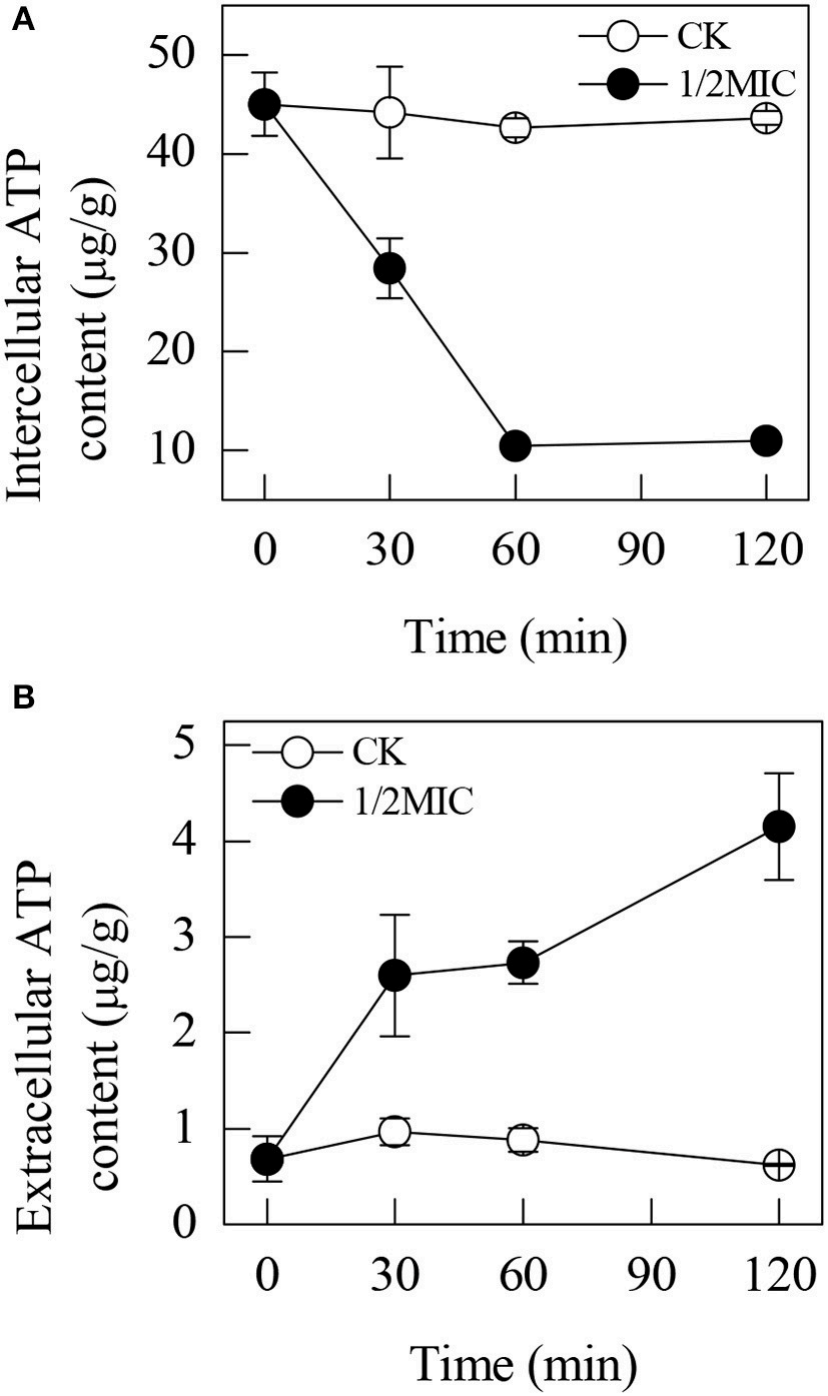
Effects of citral on the intracellular ATP content **(A)** and extracellular content **(B)** of *P. digitatum* mycelia. Data presented are the means of the pooled data. Error bars indicate the SDs of the means (*n* = 3).

The intracellular ATP contents in the *P. digitatum* cells treated with citral continuously decreased during the entire period, whereas those in the untreated cells remained stable (Figure 3A). After incubation with 1/2MIC of citral for 30 min, the intracellular ATP content was 28.4 ± 3.0 μg/g, which was lower than that of the control (44.2 ± 4.6 μg/g).

Citral exhibited an opposite effect on the extracellular ATP content (Figure 3B). After 30 min of exposure, the extracellular ATP content in the control suspensions (1.0 ± 0.1 μg/g) was lower than those treated with 1/2MIC treatments (2.6 ± 0.6 μg/g). As the culture time increased, the extracellular ATP content sharply increased. At 120 min of exposure, the extracellular ATP content in the *P. digitatum* cells treated with 1/2MIC citral was 4.2 ± 0.55 μg/g, which was still much higher than that of the control samples (0.6 ± 0.0 μg/g).

### MMP

**Figure 4:**
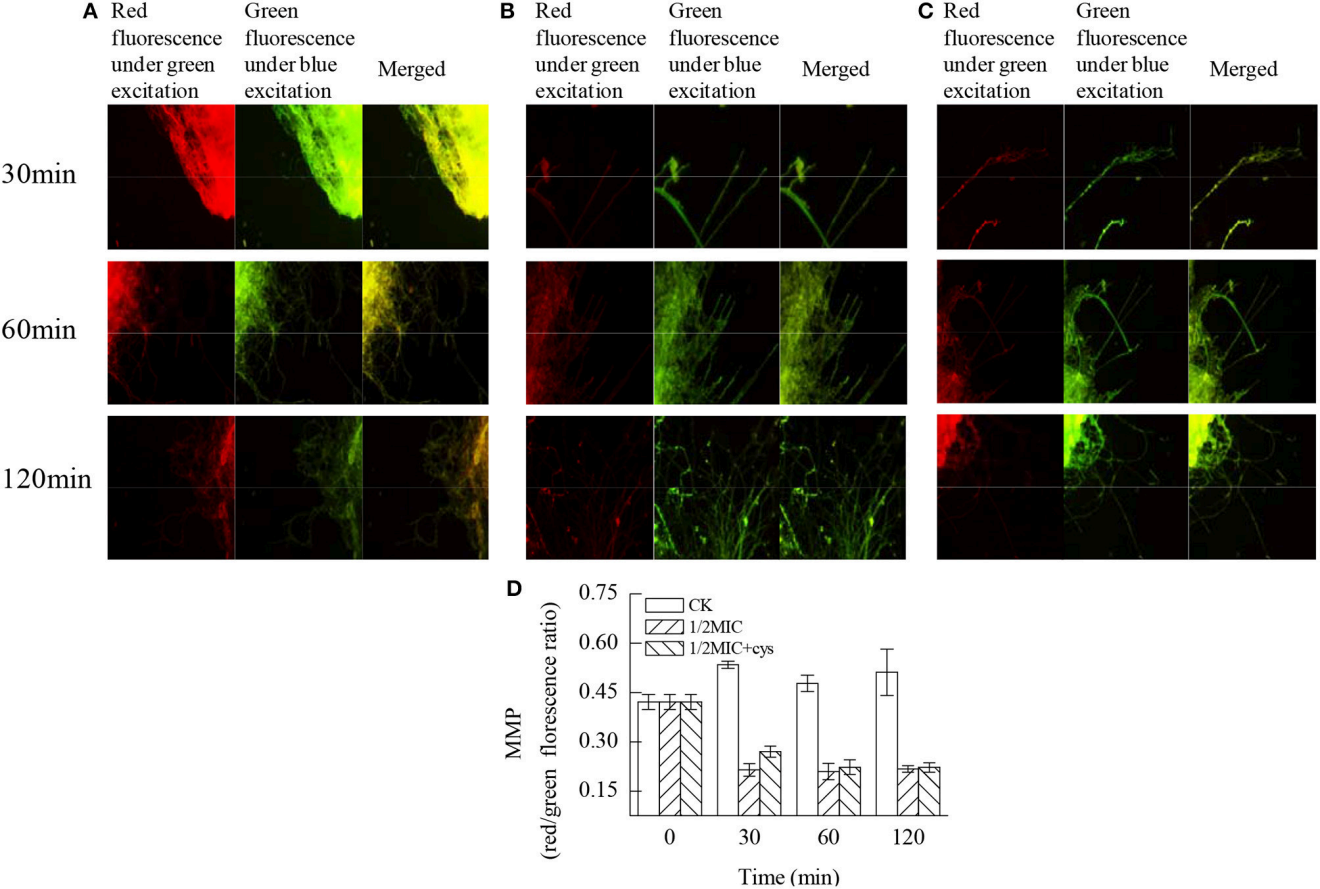
Effects of citral on the MMP of *P. digitatum* mycelia. **(A)** CK group treated for 30, 60, and 120 min; **(B)** 1/2MIC group treated for 30, 60, and 120 min; **(C)** 1/2MIC + Cys group treated for 30, 60, and 120 min; **(D)** mycelia fluorescence times. Data presented are the means of the pooled data. Error bars indicate the SDs of the means (*n* = 3).

According to the data in Figure 4, citral induced an immediate decrease on the MMP (*P* < 0.05). In untreated cells, the red/green fluorescence ratio was 0.534 ± 0.011 at 30 min. However, the addition of citral caused an immediate loss of MMP, blocking JC-10 entry to the mitochondria, leaving the JC-10 monomers to fluoresce green within the cytoplasm. This finding was reflected in the red/green fluorescence ratio, which decreased to 0.214 ± 0.019 following citral treatment for 30 min and was significantly lower than that of the control sample (*P* < 0.05). Moreover, the addition of cysteine (Cys) could delay the reduction of MMP to a certain degree, with a red/green fluorescence ratio of 0.270 ± 0.017 at 30 min, which was significantly higher (*P* < 0.05) than that in the citral treatment (Figure 4C).

### ROS levels

**Figure 5:**
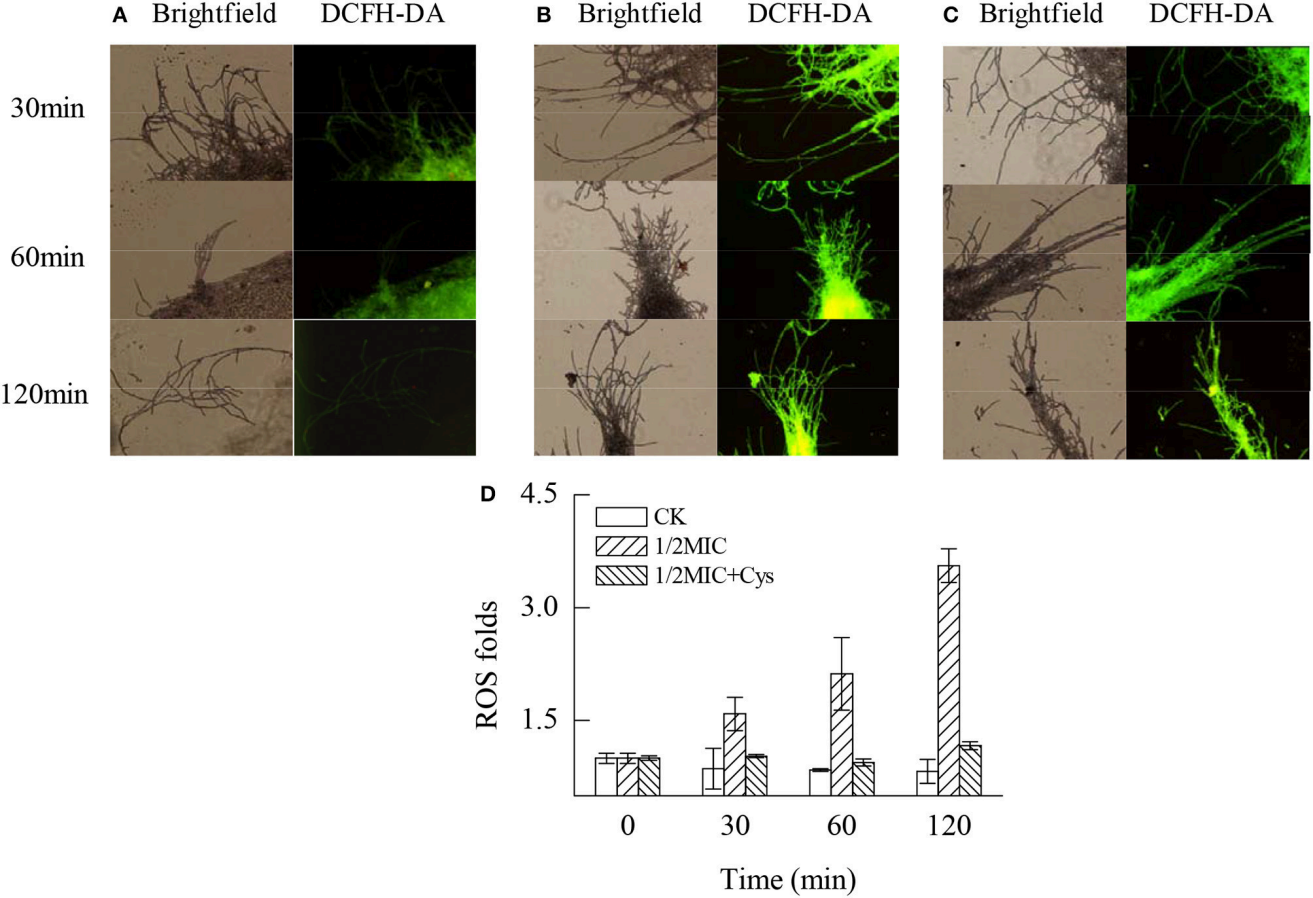
Effects of citral on the ROS accumulation of *P. digitatum* mycelia. **(A)** CK group treated for 30, 60, and 120 min; **(B)** 1/2MIC group treated for 30, 60, and 120 min; **(C)** 1/2MIC + Cys group treated for 30, 60, and 120 min; **(D)** mycelia fluorescence times. Data presented are the means of the pooled data. Error bars indicate the SDs of the means (*n* = 3).

As illustrated by Figure 5, citral treatment significantly induced the massive accumulation of ROS in the *P. digitatum* mycelia (*P* < 0.05). After 120 min of exposure, the ROS levels in the *P. digitatum* mycelia treated with 1/2MIC of citral were 3.55-fold higher than that of the control (Figure 5D). In contrast, the ROS accumulation in *P. digitatum* cells induced by citral treatment was evidently repressed (*P* < 0.05) by the addition of Cys. These results were consistent with the results of the fluorescence microscopy (Figures 5A–C).

### Plasma membrane integrity

**Figure 6:**
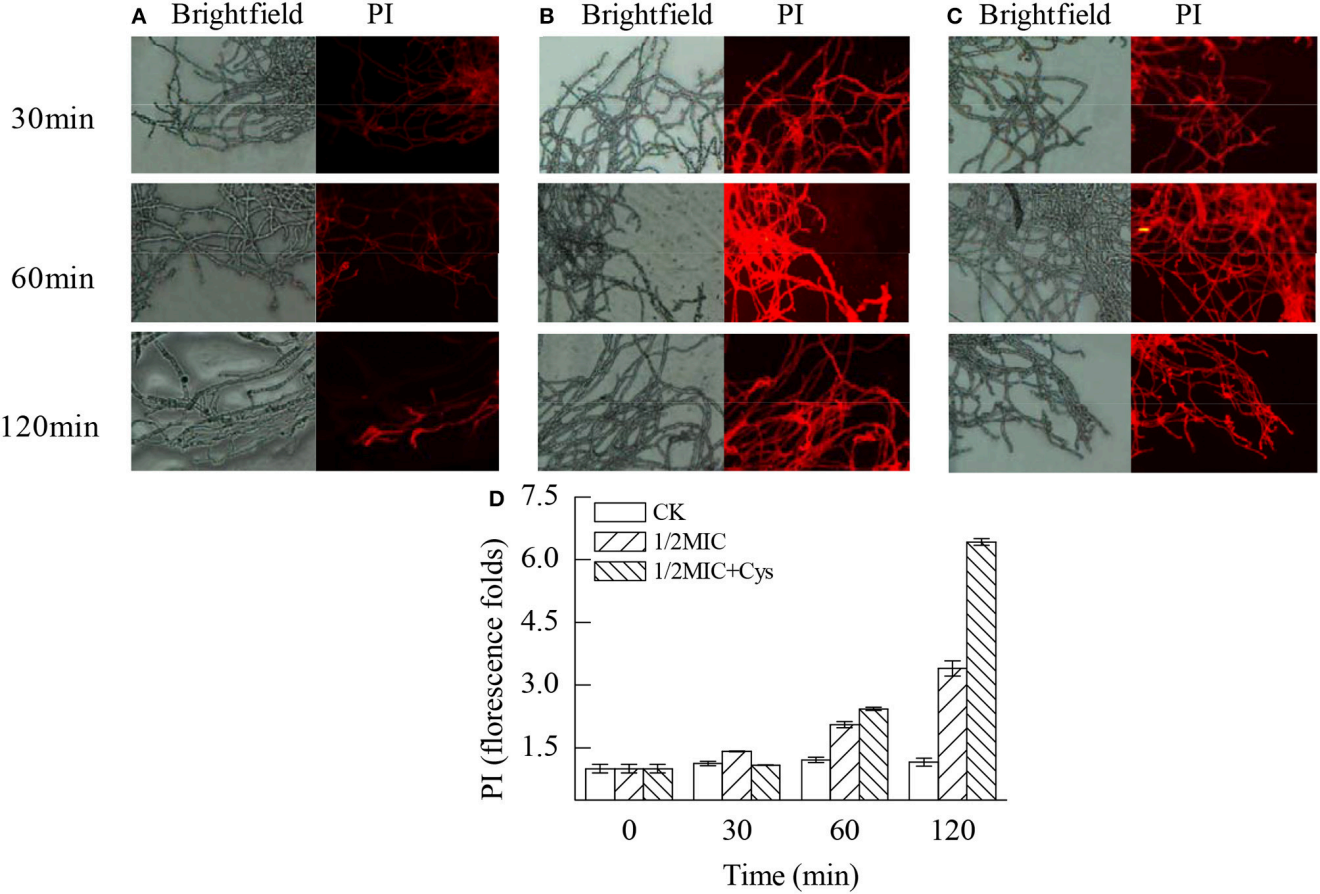
Effects of citral on the plasma membrane integrity of *P. digitatum* mycelia. **(A)** CK group treated for 30, 60, and 120 min; **(B)** 1/2MIC group treated for 30, 60, and 120 min; **(C)** 1/2MIC + Cys group treated for 30, 60, and 120 min; **(D)** mycelia fluorescence times. Data presented are the means of the pooled data. Error bars indicate the SDs of the means (*n* = 3).

The plasma membrane integrity of *P. digitatum* was markedly damaged by citral (*P* < 0.05; Figure 6). As revealed by Figure 6A, a slight red fluorescence was observed in the control hyphae. In contrast, the hyphae in Figure 6B had a strong red fluorescence. These results were consistent with the result of the fluorescence spectrophotometer that the fluorescence intensity of the 1/2MIC group was 3.4 times higher than the control groups after 120 min treatment (Figure 6D, *P* < 0.05). Before treatment with 1/2MIC of citral + Cys for 30 min, hyphae exhibited relatively slight red fluorescence, indicating that the exposure to citral + Cys induced the permeation of PI in fewer cells (Figure 6C). After 60 min, the hyphae in the treatment group showed markedly higher (*P* < 0.05) staining intensity than that of the control group (Figure 6D).

### Effect of exogenous cys on the antifungal activity of citral against *P. digitatum*

The antifungal activity of citral against *P. digitatum* cells was alleviated by the addition of Cys (Table 3). After 2 days of culture with Cys, only 30.2 and 19.05% of the mycelial growths were inhibited by MIC and 1/2 MIC of citral, respectively. As similar phenomenon was also observed after 4 days of culture.

**Table 3:** Effect of exogenous Cys on the antifungal activity of citral against *P. digitatum*.

**Compound**	**Antifungal rate (%)**			
	**1d**	**2d**	**3d**	**4d**
1.0 μL/mL citral	37.5 ± 0.0b	55.1 ± 0.0b	16.1 ± 2.0c	19.2 ± 1.4b
1.0 μL/mL citral + Cys	25.0 ± 0.0c	19.05 ± 0.0d	9.7 ± 0.0d	8.4 ± 1.0d
2.0 μL/mL citral	100.0 ± 0.0a	100.0 ± 0.0a	32.2 ± 2.0a	42.9 ± 0.7a
2.0 μL/mL citral + Cys	39.3 ± 6.2b	30.2 ± 3.6c	23.0 ± 2.7b	10.5 ± 0.0c

*a–d Significant differences at P < 0.05 according to Duncan's multiple range test. Values are presented as the mean ± SD*.

## Discussions

In the present study, a comprehensive proteome analysis was determined to study the antifungal mechanism of citral against *P. digitatum*. A total of 82 DEPs were identified in 1/2MIC citral-treated samples. These DEPs were mainly involved in oxidative phosphorylation, the TCA cycle, glycolysis and translationally related pathways, ribosome biogenesis in eukaryotes, the mRNA surveillance pathway, and RNA transport (Table 1), which were consistent with our previous results obtained by RNA-Seq analysis (OuYang et al., 2016b).

Oxidative phosphorylation is the primary source of the energy-producing pathway in eukaryotic cells, which is catalyzed by five mitochondrial complexes (I–V) (Chaban et al., 2014). A recent study has revealed that the antifungal activity of garlic oil against *C. albicans* was attributed to the severe disruption of oxidative phosphorylation (Li W. R. et al., 2016). In the current study, 10 DEPs involved in oxidative phosphorylation were obtained after citral treatment (Table 2). Among of these DEPs, three subunits of mitochondrial complex I (Ndufb9, Ndufs6, and Ndufb8), mitochondrial complex III (QCR6), and two subunits of mitochondrial complex IV (COX6B and COX17), were all down-regulated. In contrast, one subunit of mitochondrial complex II (SDHC) and QCR10 and the ATP synthase delta chain as well as the subunits belonging to the mitochondrial complex III and mitochondrial complex V, respectively, were all up-regulated. To confirm this finding, the enzymatic activities of the above five mitochondrial complex enzymes were further measured. After exposure to citral, the activities of mitochondrial complex I and complex IV were inhibited, whereas the activities of mitochondrial complex II, complex III and complex V were significantly induced (*P* < 0.05, Figure 1). These results were largely consistent with the iTRAQ results. It should be pointed out, however, that the DEPs comprising the mitochondrial complexes III exhibited an opposite expression pattern irrespective of the increased enzymatic activity of mitochondrial complexes III. This phenomenon could be explained by the fact that the enzymatic activities of proteins are generally determined by the coordination of different subunits (Buechler et al., 1991).

Mitochondrial oxidative phosphorylation constitutes the major cellular ATP-producing mechanism under aerobic conditions and hence plays an important role in maintaining the ATP levels in the cell. The function of oxidative phosphorylation is to synthesize ATP by generating a proton gradient within the inner mitochondrial membrane. Blocking or restraining oxidative phosphorylation can effectively decrease the ATP concentrations in the cell (Wang et al., 2015). In another study, the inhibitor of mitochondrial electron transport could reduce MMP via inhibiting the proton-pumping function of the respiratory chain, leading to the reduction of ATP production and cell death (Kaim and Dimroth, 1999). In this study, a decrease in the content of intracellular ATP and an increase in the content of extracellular ATP were observed. Moreover, exposure to citral led to a significant decrease in the MMP (Figure 4). These observations indicated the existence of irreversible mitochondrial membrane damage, which would consequently lead to an efflux of ATP from the collapsed mitochondrial membrane and an increase in the extracellular ATP content. These results were also in agreement with our previous observations that citral treatment could lead to the morphological changes of mitochondria, the reduction of the ATP content and the inhibition of the TCA cycle in *P. digitatum* hyphae (Zheng et al., 2015). Similar results were reported in some earlier studies (Machado et al., 2012; Xia et al., 2013). It is worth noticing that the oxidative damage of mitochondrial proteins and the collapse of the MMP were generally supposed to be the result of undesirable accumulation of ROS, and the accumulation of ROS might affect the normal morphology and function of mitochondria (Genova et al., 2004; Fujita et al., 2014; Tian et al., 2016). Therefore, the above results indicated that the abnormal leakage of electrons from the mitochondrial respiratory chain might be caused by oxidative damage in fungal cells.

In fact, the mitochondrial respiratory chain is a major source of ROS (Tian et al., 2013). This process was highly regulated by mitochondrial complex I, complex II, and complex III (Finkel and Holbrook, 2000; Tian et al., 2013). In addition, the inhibition of mitochondrial complex IV could lead to the incompletely catalysis of oxygen, resulting in the generation of ROS through mitochondrial complex I or complex III (Semenza, 2007). Previous studies have demonstrated that the antifungal action of some essential oils, such as dill oil, *Curcuma longa* oil and thymol, were positively related with the accumulation of ROS (Tian et al., 2012; Kumar et al., 2016; Shen et al., 2016). Similarly, higher fluorescence values were exclusively observed in the 1/2MIC citral treated samples. This phenomenon was obvious with the increasing of the exposure time (Figure 5). This result is consistent with our previous study suggesting that citral could induce the massive accumulation of H_2_O_2_ in *P. digitatum* and lead to lipid peroxidation via oxidation burst (OuYang et al., 2016a).

Normally, the balance between ROS production and antioxidant defenses determines the degree of oxidative stress. Our previous study found that the addition of citral resulted in oxidative stress by stimulating the activities of lipoxygenase and peroxidase in *P. digitatum* (OuYang et al., 2016a). In this study, the up-regulation of GST, a key enzyme involved in glutathione metabolism, also supported this point of view, whose activity was significantly increased after the addition of citral (*P* < 0.05; Figure 2A). The significant decrease in the GSH content (*P* < 0.05; Figure 2B) further confirmed this hypothesis. In a previous study, citral was illustrated to be able to stimulate the activity of GST in RL34 cells, whereas geranial treatment could attenuate the intracellular GSH level, and this process was accompanied with the increasing ROS content (Nakamura et al., 2003). Similar results were also reported in some other investigations (Guha et al., 2011; Pramanik et al., 2011). These results again suggested that citral addition could lead to the oxidative damage of *P. digitatum* hyphae.

It is generally accepted that the accumulation of ROS will result in the breakdown of the normal cellular, membrane and reproductive functions by oxidizing lipids, proteins, nucleic acids, and carbohydrates (Qin et al., 2007; Tian et al., 2012, 2013). In this study, the plasma membrane integrity was also positively correlated with the accumulation of ROS. As revealed by Figure 6, exposure to 1/2MIC of citral apparently induced a severe plasma membrane lesion, as convinced by the results of PI staining. Accordingly, this process was accompanied by the massive accumulation of ROS (Figure 5).

Application of exogenous antioxidants could maintain ROS at the basal level and repair cellular damage caused by ROS (Lai et al., 2011; Tian et al., 2013). Liu et al. (2013) reported that the addition of antioxidant Cys could significantly reduce the detrimental effects of D-limonene on *S. cerevisiae*. To further confirm the oxidative damage of *P. digitatum* hyphae, Cys was added to the culture media. Cys is the rate-limiting precursor for the synthesis of GSH and is also the preeminent antioxidant of the cell (Tian et al., 2013). Cys could prevent the accumulation of ROS and alleviate the oxidative damage of cells. As shown in Table 3, addition of exogenous Cys significantly reduced the antifungal activity of citral against *P. digitatum*. In addition, Cys maintained the basal ROS level (Figure 5), and deferred the decrease of MMP (Figure 4) and the membrane damage in citral treated samples (Figure 6D). These results indicated that ROS might serve as a mediator in regulating the antifungal activity of citral against *P. digitatum*.

Taken together, our present study suggests that the antifungal activity of citral against *P. digitatum* is caused by damaged oxidative phosphorylation through massive ROS accumulation. These findings not only provide a better understanding of antifungal mechanism of plant essential oils but also provide important theoretical guidance for the development of novel fungicides, reducing the postharvest decay of fruits in the future.

## Author contributions

NT designed research; QO performed research; NT and QO analyzed data; QO, NT and MZ wrote the paper. All authors contributed to study design and provided input on the manuscript preparation. All authors have given approval to the final version of the manuscript.

### Conflict of interest statement

The authors declare that the research was conducted in the absence of any commercial or financial relationships that could be construed as a potential conflict of interest.

## Abbreviations

DEPs: Differentially expressed proteins
iTRAQ: isobaric tags for relative and absolute quantization
MMP: mitochondrial membrane potential
ROS: reactive oxygen species
Cys: cysteine
GRAS: Generally Recognized as Safe
MFC: minimum antifungal concentration
MIC: minimum inhibitory concentration
TCA cycle: citrate cycle
PDB: potato dextrose broth
TEAB: triethylammonium bicarbonate
KEGG: Kyoto Encyclopedia of Genes and Genomes
GST: glutathione S-transferase
GSH: glutathione
DCFH-DA: redox-sensitive fluorescent probe dichloro-dihydro-fluorescein diacetate
PI: propidium iodide.
